# Flunarizinium isonicotinate

**DOI:** 10.1107/S1600536814010423

**Published:** 2014-05-17

**Authors:** Channappa N. Kavitha, Manpreet Kaur, Jerry P. Jasinski, H. S. Yathirajan

**Affiliations:** a Department of Studies in Chemistry, University of Mysore, Manasagangotri, Mysore 570 006, India; b Department of Chemistry, Keene State College, 229 Main Street, Keene, NH 03435-2001, USA

## Abstract

In the cation of the title salt {systematic name: 4-[bis­(4-fluoro­phen­yl)meth­yl]-1-[(2*E*)-3-phenyl­prop-2-en-1-yl]piperazin-1-ium pyridine-4-carboxyl­ate}, C_26_H_27_F_2_N_2_^+^·C_6_H_4_NO_2_^−^, the piperazine ring is in a slightly distorted chair conformation. The dihedral angle between the mean planes of the fluoro-substituted benzene rings is 81.9 (1)° and these benzene rings form dihedral angles of 6.5 (1) and 87.8 (1)° with the phenyl ring. In the crystal, a single N—H⋯O hydrogen bond links the cation and the anion. In addition, weak C—H⋯O hydrogen bonds and π–π stacking inter­actions involving one of the fluoro-substituted benzene rings and the phenyl ring, with a centroid–centroid distance of 3.700 (7) Å, link mol­ecules along [100].

## Related literature   

For the bioligical activities of flunarizine, see: Amery (1983 ▶); Holmes *et al.* (1984 ▶). For related structures, see: Kavitha *et al.* (2013*a* ▶,*b* ▶,*c* ▶,*d* ▶). For puckering parameters, see Cremer & Pople (1975 ▶). For standard bond lengths, see: Allen *et al.* (1987 ▶).
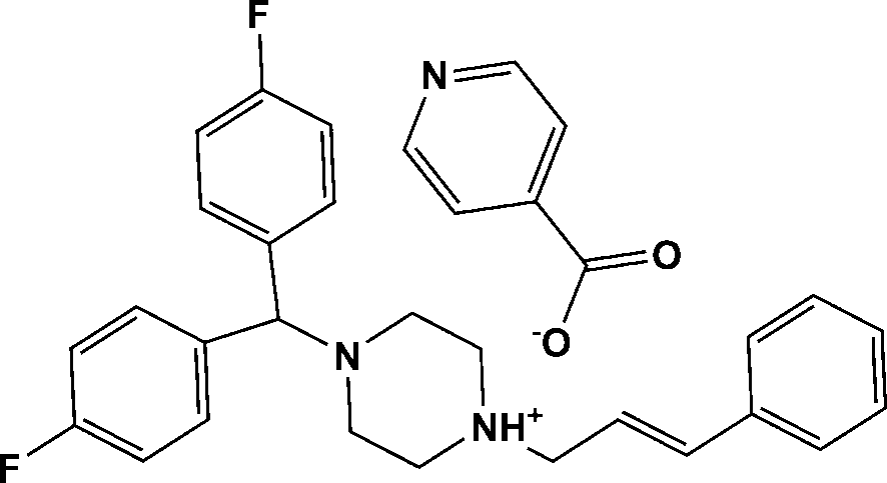


## Experimental   

### 

#### Crystal data  

C_26_H_27_F_2_N_2_^+^·C_6_H_4_NO_2_^−^
*M* *_r_* = 527.60
Monoclinic, 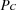

*a* = 11.0023 (3) Å
*b* = 10.6435 (3) Å
*c* = 11.3393 (3) Å
β = 92.481 (3)°
*V* = 1326.63 (6) Å^3^
*Z* = 2
Cu *K*α radiation
μ = 0.76 mm^−1^
*T* = 173 K
0.22 × 0.12 × 0.06 mm

#### Data collection  

Agilent Eos Gemini diffractometer
Absorption correction: multi-scan (*CrysAlis PRO* and *CrysAlis RED*; Agilent, 2012 ▶) *T*_min_ = 0.781, *T*_max_ = 1.000
8232 measured reflections
3403 independent reflections
3238 reflections with *I* > 2σ(*I*)
*R*_int_ = 0.042

#### Refinement  

*R*[*F* ^2^ > 2σ(*F* ^2^)] = 0.040
*wR*(*F* ^2^) = 0.110
*S* = 1.02
3403 reflections
357 parameters
2 restraints
H atoms treated by a mixture of independent and constrained refinement
Δρ_max_ = 0.18 e Å^−3^
Δρ_min_ = −0.20 e Å^−3^
Absolute structure: Flack (1983 ▶), 857 Friedel pairs
Absolute structure parameter: 0.2 (2)

### 

Data collection: *CrysAlis PRO* (Agilent, 2012 ▶); cell refinement: *CrysAlis PRO*; data reduction: *CrysAlis RED* (Agilent, 2012 ▶); program(s) used to solve structure: *SUPERFLIP* (Palatinus & Chapuis, 2007 ▶); program(s) used to refine structure: *SHELXL2012* (Sheldrick, 2008 ▶); molecular graphics: *OLEX2* (Dolomanov *et al.*, 2009 ▶) and *PLATON* (Spek, 2009 ▶); software used to prepare material for publication: *OLEX2*.

## Supplementary Material

Crystal structure: contains datablock(s) I. DOI: 10.1107/S1600536814010423/lh5702sup1.cif

Structure factors: contains datablock(s) I. DOI: 10.1107/S1600536814010423/lh5702Isup2.hkl

Supporting information file. DOI: 10.1107/S1600536814010423/lh5702Isup3.cml

CCDC reference: 1001665

Additional supporting information:  crystallographic information; 3D view; checkCIF report

## Figures and Tables

**Table 1:** Hydrogen-bond geometry (Å, °)

*D*—H⋯*A*	*D*—H	H⋯*A*	*D*⋯*A*	*D*—H⋯*A*
N2*A*—H2*A*⋯O1*B* ^i^	0.94 (4)	1.62 (4)	2.557 (3)	176 (4)
C6*A*—H6*A*⋯O2*B* ^ii^	0.93	2.60	3.439 (4)	151

Symmetry codes: (i) 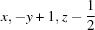; (ii) 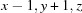.

**Fig. 1.**
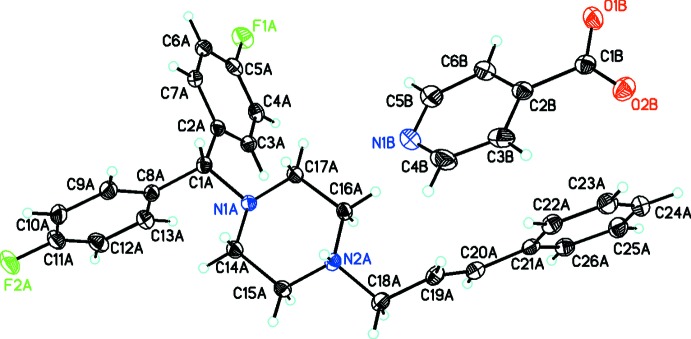
The asymmetric unit of (I) showing 30% probability displacement ellipsoids.

**Fig. 2.**
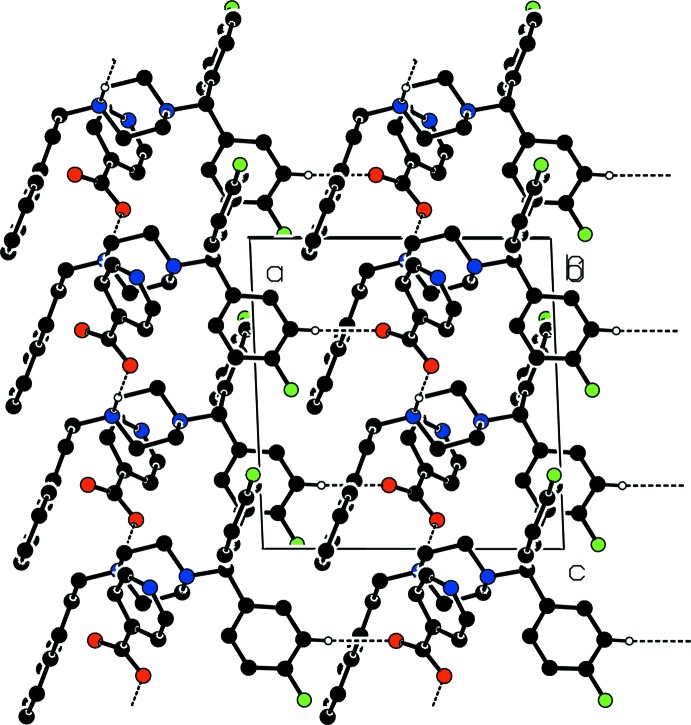
Part of the crystal structure of (I) with hydrogen bonds shown as dashed lines. H atoms not involved in hydrogen bonds have been removed for clarity.

## supplementary crystallographic information

### 1. Comment

Flunarizine (1-[bis(4-fluorophenyl)methyl]-4-[(2E)-3-phenylprop-2-en-1-yl]piperazine), a piperazine derivative is a non-selective calcium antagonist (Amery, 1983). It is effective in the prophylaxis of migraine, occlusive peripheral vascular disease, vertigo of central and peripheral origin, and as an adjuvant in the therapy of epilepsy. A review of its pharmacodynamic and pharmacokinetic properties and therapeutic use is published (Holmes *et al.*, 1984). The crystal structures of 4- [bis(4-fluorophenyl)methyl]-1-[(2E)-3-phenylprop-2-en-1-yl]piperazin-1-ium- 3-carboxy propanoate (Kavitha *et al.*, 2013*a*), flunarizinium hydrogen maleate (Kavitha *et al.*, 2013*b*), cinnarizinium fumarate (Kavitha *et al.*, 2013*c*) and cinnarizinium bis(p-toluenesulfonate)dihydrate (Kavitha *et al.*, 2013*d*) have been reported. In view of the importance of flunarizine, this paper reports the crystal structure study of flunarizinium isonicotinate, (I), C_26_H_27_F_2_N_2_^+^.C_6_H_4_NO_2_^-^.

The title salt, (I), crystallizes with one piperazinium cation (A) and one isonicotinate anion (B) in the asymmetric unit (Fig. 1). In the cation, the piperazine ring is in a slightly distorted chair conformation (puckering parameters Q, θ, and φ = 0.596 (3)Å, 3.3 (3)° and 10 (4)°, respectively (Cremer & Pople, 1975) and is twisted from the mean plane of the phenyl ring in an anti-periplanar conformation with a torsion angle of -178.9 (7)°. The dihedral angle between the mean planes of the two fluoro-substituted benzene rings is 81.9 (1)°. Of the two fluro-substitutted benzene ring rings, one (C8A–C13A) is almost planar with respect to the mean plane of the phenyl ring forming a diherdral angle of 6.5 (1)°, while the other (C2A–C7A) is twisted by 87.8 (1)°. Bond lengths are in normal ranges (Allen *et al.*, 1987). A single N2A—H2A···O1B^i^ intermolecular hydrogen bond links the cation with the anion (Fig. 2). In addition, weak C—H···O intermolecular interactions (Table 1) and a weak π–π stacking interaction involving one of the fluoro-substituted benzene rings and the phenyl ring, link the molecules along [100] (Cg1–Cg2 = 3.700 (7)Å; -1+x, 1+y, z; Cg1 = C8A–C13A; Cg2 = C21A–C26A).

### 2. Experimental

Flunarizine (2.025 g, 0.01 mol) and isonicotinic acid (0.61 g, 0.005 mol) were dissolved in hot dimethylformamide solution and stirred over a magnetic stirrer for 10 minutes. The resulting solution was allowed to cool slowly at room temperature. The crystals of the title compound appeared after a few days and were subsequently used for x-ray studies.

### 3. Refinement

Atom H2A was refined isotropically and the remaining H atoms were placed in calculated positions and then refined using a riding-model approximation with C—H = 0.93Å or 0.98Å(CH) or 0.97Å (CH_2_). Isotropic displacement parameters for these atoms were set to 1.2 (CH, CH_2_) times *U*_eq_ of the parent atom.

### Crystal data

**Table:**

C_26_H_27_F_2_N_2_^+^·C_6_H_4_NO_2_^−^	*F*(000) = 556
*M**_r_* = 527.60	*D*_x_ = 1.321 Mg m^−^^3^
Monoclinic, *P**c*	Cu *K*α radiation, λ = 1.54184 Å
*a* = 11.0023 (3) Å	Cell parameters from 4115 reflections
*b* = 10.6435 (3) Å	θ = 4.0–71.5°
*c* = 11.3393 (3) Å	µ = 0.76 mm^−^^1^
β = 92.481 (3)°	*T* = 173 K
*V* = 1326.63 (6) Å^3^	Block, colourless
*Z* = 2	0.22 × 0.12 × 0.06 mm

### Data collection

**Table:**

Agilent Eos Gemini diffractometer	3403 independent reflections
Radiation source: Enhance (Cu) X-ray Source	3238 reflections with *I* > 2σ(*I*)
Detector resolution: 16.0416 pixels mm^-1^	*R*_int_ = 0.042
ω scans	θ_max_ = 71.3°, θ_min_ = 4.0°
Absorption correction: multi-scan (*CrysAlis PRO* and *CrysAlis RED*; Agilent, 2012)	*h* = −13→7
*T*_min_ = 0.781, *T*_max_ = 1.000	*k* = −12→13
8232 measured reflections	*l* = −13→13

### Refinement

**Table:**

Refinement on *F*^2^	H atoms treated by a mixture of independent and constrained refinement
Least-squares matrix: full	*w* = 1/[σ^2^(*F*_o_^2^) + (0.0764*P*)^2^ + 0.0707*P*] where *P* = (*F*_o_^2^ + 2*F*_c_^2^)/3
*R*[*F*^2^ > 2σ(*F*^2^)] = 0.040	(Δ/σ)_max_ < 0.001
*wR*(*F*^2^) = 0.110	Δρ_max_ = 0.18 e Å^−^^3^
*S* = 1.02	Δρ_min_ = −0.20 e Å^−^^3^
3403 reflections	Extinction correction: *SHELXL2012* (Sheldrick, 2008), Fc^*^=kFc[1+0.001xFc^2^λ^3^/sin(2θ)]^-1/4^
357 parameters	Extinction coefficient: 0.0050 (8)
2 restraints	Absolute structure: Classical Flack method preferred over Parsons because s.u. lower. 857 Friedel pairs
Primary atom site location: structure-invariant direct methods	Absolute structure parameter: 0.2 (2)
Hydrogen site location: mixed	

### Special details

**Table:**

Geometry. All esds (except the esd in the dihedral angle between two l.s. planes) are estimated using the full covariance matrix. The cell esds are taken into account individually in the estimation of esds in distances, angles and torsion angles; correlations between esds in cell parameters are only used when they are defined by crystal symmetry. An approximate (isotropic) treatment of cell esds is used for estimating esds involving l.s. planes.

### Fractional atomic coordinates and isotropic or equivalent isotropic displacement parameters (Å^2^)

**Table:**

	*x*	*y*	*z*	*U*_iso_*/*U*_eq_	
F1A	−0.1154 (2)	1.19666 (19)	0.98648 (18)	0.0538 (5)	
F2A	0.0248 (2)	1.5040 (2)	0.2592 (2)	0.0696 (7)	
N1A	0.2572 (2)	1.0742 (2)	0.58937 (19)	0.0263 (5)	
N2A	0.4826 (2)	0.9352 (2)	0.5718 (2)	0.0272 (5)	
H2A	0.461 (4)	0.877 (4)	0.513 (4)	0.048 (10)*	
C1A	0.1259 (2)	1.0983 (2)	0.5668 (2)	0.0270 (5)	
H1A	0.0897	1.0231	0.5298	0.032*	
C2A	0.0641 (2)	1.1206 (2)	0.6827 (2)	0.0271 (5)	
C3A	0.1256 (3)	1.1748 (2)	0.7798 (2)	0.0291 (5)	
H3A	0.2080	1.1932	0.7763	0.035*	
C4A	0.0650 (3)	1.2017 (2)	0.8823 (3)	0.0336 (6)	
H4A	0.1062	1.2378	0.9472	0.040*	
C5A	−0.0561 (3)	1.1741 (3)	0.8856 (3)	0.0362 (6)	
C6A	−0.1212 (3)	1.1206 (3)	0.7915 (3)	0.0375 (7)	
H6A	−0.2037	1.1033	0.7959	0.045*	
C7A	−0.0596 (3)	1.0937 (3)	0.6905 (3)	0.0323 (6)	
H7A	−0.1016	1.0569	0.6264	0.039*	
C8A	0.1019 (2)	1.2089 (2)	0.4835 (2)	0.0286 (5)	
C9A	0.0496 (3)	1.1870 (3)	0.3720 (3)	0.0350 (6)	
H9A	0.0320	1.1052	0.3482	0.042*	
C10A	0.0231 (3)	1.2866 (4)	0.2952 (3)	0.0441 (8)	
H10A	−0.0122	1.2723	0.2204	0.053*	
C11A	0.0504 (3)	1.4057 (3)	0.3327 (3)	0.0460 (8)	
C12A	0.1034 (3)	1.4311 (3)	0.4423 (3)	0.0454 (8)	
H12A	0.1216	1.5132	0.4649	0.054*	
C13A	0.1292 (3)	1.3318 (3)	0.5183 (3)	0.0366 (6)	
H13A	0.1649	1.3471	0.5927	0.044*	
C14A	0.3274 (2)	1.0792 (3)	0.4828 (2)	0.0301 (6)	
H14A	0.3156	1.1601	0.4446	0.036*	
H14B	0.2991	1.0145	0.4281	0.036*	
C15A	0.4611 (3)	1.0600 (3)	0.5144 (2)	0.0297 (6)	
H15A	0.5070	1.0650	0.4434	0.036*	
H15B	0.4895	1.1261	0.5676	0.036*	
C16A	0.4061 (2)	0.9246 (2)	0.6769 (2)	0.0280 (5)	
H16A	0.4332	0.9853	0.7362	0.034*	
H16B	0.4148	0.8413	0.7108	0.034*	
C17A	0.2741 (3)	0.9485 (2)	0.6415 (2)	0.0286 (5)	
H17A	0.2463	0.8855	0.5847	0.034*	
H17B	0.2253	0.9412	0.7104	0.034*	
C18A	0.6153 (3)	0.9189 (3)	0.6037 (3)	0.0338 (6)	
H18A	0.6603	0.9180	0.5321	0.041*	
H18B	0.6435	0.9900	0.6508	0.041*	
C19A	0.6416 (3)	0.8002 (3)	0.6713 (3)	0.0328 (6)	
H19A	0.6247	0.7235	0.6349	0.039*	
C20A	0.6877 (3)	0.8008 (3)	0.7806 (3)	0.0324 (6)	
H20A	0.7019	0.8796	0.8139	0.039*	
C21A	0.7198 (2)	0.6926 (3)	0.8567 (2)	0.0306 (6)	
C22A	0.7056 (3)	0.5679 (3)	0.8189 (3)	0.0360 (6)	
H22A	0.6718	0.5512	0.7440	0.043*	
C23A	0.7412 (3)	0.4696 (3)	0.8918 (3)	0.0420 (7)	
H23A	0.7306	0.3872	0.8658	0.050*	
C24A	0.7929 (3)	0.4928 (3)	1.0041 (3)	0.0439 (7)	
H24A	0.8182	0.4263	1.0523	0.053*	
C25A	0.8064 (3)	0.6152 (3)	1.0436 (3)	0.0420 (7)	
H25A	0.8398	0.6314	1.1188	0.050*	
C26A	0.7696 (3)	0.7141 (3)	0.9699 (3)	0.0353 (6)	
H26A	0.7785	0.7963	0.9969	0.042*	
O1B	0.4163 (2)	0.2192 (2)	0.9087 (2)	0.0479 (6)	
O2B	0.5711 (2)	0.1759 (2)	0.7956 (2)	0.0434 (5)	
N1B	0.3838 (3)	0.5717 (3)	0.6228 (4)	0.0636 (10)	
C1B	0.4838 (3)	0.2391 (3)	0.8226 (2)	0.0326 (6)	
C2B	0.4475 (3)	0.3537 (3)	0.7500 (2)	0.0328 (6)	
C3B	0.4993 (3)	0.3812 (3)	0.6435 (3)	0.0438 (7)	
H3B	0.5574	0.3281	0.6132	0.053*	
C4B	0.4628 (4)	0.4892 (4)	0.5830 (3)	0.0604 (11)	
H4B	0.4956	0.5046	0.5102	0.072*	
C5B	0.3354 (4)	0.5444 (4)	0.7234 (4)	0.0584 (10)	
H5B	0.2787	0.6003	0.7518	0.070*	
C6B	0.3624 (3)	0.4388 (3)	0.7899 (3)	0.0401 (7)	
H6B	0.3244	0.4246	0.8603	0.048*	

### Atomic displacement parameters (Å^2^)

**Table:**

	*U*^11^	*U*^22^	*U*^33^	*U*^12^	*U*^13^	*U*^23^
F1A	0.0610 (13)	0.0588 (12)	0.0435 (11)	0.0125 (10)	0.0239 (9)	0.0020 (9)
F2A	0.0701 (15)	0.0701 (14)	0.0688 (15)	0.0143 (11)	0.0076 (12)	0.0431 (12)
N1A	0.0251 (11)	0.0285 (10)	0.0256 (11)	0.0021 (8)	0.0031 (8)	0.0020 (8)
N2A	0.0258 (12)	0.0318 (11)	0.0238 (11)	0.0027 (9)	−0.0002 (8)	−0.0024 (9)
C1A	0.0282 (13)	0.0253 (12)	0.0272 (13)	−0.0001 (10)	−0.0023 (10)	−0.0015 (10)
C2A	0.0277 (13)	0.0240 (12)	0.0296 (13)	0.0034 (10)	0.0017 (10)	0.0034 (9)
C3A	0.0301 (13)	0.0258 (12)	0.0316 (13)	0.0011 (10)	0.0017 (10)	0.0002 (9)
C4A	0.0445 (16)	0.0266 (12)	0.0295 (13)	0.0066 (11)	0.0006 (12)	−0.0001 (10)
C5A	0.0448 (17)	0.0306 (13)	0.0345 (14)	0.0099 (12)	0.0149 (12)	0.0086 (11)
C6A	0.0300 (14)	0.0377 (15)	0.0452 (17)	0.0050 (12)	0.0074 (12)	0.0109 (12)
C7A	0.0305 (14)	0.0316 (13)	0.0342 (14)	0.0005 (10)	−0.0029 (11)	0.0066 (11)
C8A	0.0257 (12)	0.0324 (13)	0.0277 (13)	0.0044 (11)	0.0021 (10)	0.0024 (10)
C9A	0.0313 (14)	0.0428 (15)	0.0309 (14)	0.0020 (11)	0.0016 (11)	0.0018 (12)
C10A	0.0341 (16)	0.067 (2)	0.0311 (15)	0.0048 (14)	−0.0008 (12)	0.0123 (14)
C11A	0.0438 (18)	0.0467 (18)	0.0480 (19)	0.0079 (14)	0.0085 (14)	0.0221 (15)
C12A	0.0536 (19)	0.0324 (15)	0.0510 (19)	0.0035 (14)	0.0122 (15)	0.0096 (13)
C13A	0.0435 (17)	0.0324 (15)	0.0340 (15)	0.0027 (12)	0.0029 (12)	0.0029 (11)
C14A	0.0310 (14)	0.0338 (13)	0.0255 (13)	0.0031 (11)	0.0032 (11)	0.0035 (10)
C15A	0.0310 (14)	0.0338 (13)	0.0243 (13)	−0.0010 (11)	0.0036 (10)	0.0020 (10)
C16A	0.0305 (14)	0.0298 (12)	0.0238 (12)	0.0038 (10)	0.0019 (10)	0.0026 (10)
C17A	0.0293 (13)	0.0290 (12)	0.0277 (13)	0.0011 (10)	0.0032 (10)	0.0037 (10)
C18A	0.0260 (14)	0.0421 (15)	0.0332 (15)	0.0021 (11)	0.0007 (11)	−0.0016 (11)
C19A	0.0269 (14)	0.0351 (14)	0.0363 (15)	0.0047 (11)	0.0005 (11)	−0.0066 (11)
C20A	0.0276 (13)	0.0329 (14)	0.0368 (14)	−0.0007 (11)	0.0023 (11)	−0.0035 (11)
C21A	0.0220 (12)	0.0361 (14)	0.0338 (15)	−0.0008 (10)	0.0030 (10)	0.0008 (11)
C22A	0.0325 (15)	0.0396 (15)	0.0357 (15)	−0.0031 (12)	−0.0024 (12)	−0.0045 (12)
C23A	0.0389 (17)	0.0326 (14)	0.0545 (19)	0.0005 (13)	0.0029 (14)	−0.0015 (13)
C24A	0.0395 (17)	0.0445 (17)	0.0476 (18)	0.0031 (13)	0.0013 (14)	0.0131 (14)
C25A	0.0391 (16)	0.0529 (18)	0.0338 (14)	0.0006 (14)	−0.0025 (12)	0.0029 (13)
C26A	0.0339 (15)	0.0368 (15)	0.0351 (15)	−0.0031 (12)	0.0015 (12)	−0.0028 (11)
O1B	0.0465 (13)	0.0533 (13)	0.0444 (13)	0.0075 (11)	0.0073 (10)	0.0189 (10)
O2B	0.0362 (12)	0.0411 (11)	0.0529 (13)	0.0060 (9)	0.0027 (10)	−0.0053 (9)
N1B	0.0457 (18)	0.068 (2)	0.077 (2)	−0.0050 (15)	−0.0011 (16)	0.0407 (18)
C1B	0.0317 (14)	0.0346 (14)	0.0311 (13)	−0.0056 (12)	−0.0023 (11)	−0.0033 (11)
C2B	0.0324 (14)	0.0361 (14)	0.0294 (13)	−0.0091 (11)	−0.0023 (10)	−0.0008 (11)
C3B	0.0436 (17)	0.0529 (18)	0.0353 (15)	−0.0173 (14)	0.0043 (13)	−0.0030 (13)
C4B	0.057 (2)	0.087 (3)	0.0363 (18)	−0.026 (2)	−0.0027 (16)	0.0219 (18)
C5B	0.045 (2)	0.050 (2)	0.081 (3)	0.0008 (16)	0.0043 (19)	0.0200 (19)
C6B	0.0408 (17)	0.0391 (15)	0.0407 (17)	−0.0044 (13)	0.0048 (13)	0.0056 (12)

### Geometric parameters (Å, º)

**Table:**

F1A—C5A	1.363 (3)	C16A—H16A	0.9700
F2A—C11A	1.360 (4)	C16A—H16B	0.9700
N1A—C1A	1.479 (3)	C16A—C17A	1.511 (4)
N1A—C14A	1.463 (3)	C17A—H17A	0.9700
N1A—C17A	1.471 (3)	C17A—H17B	0.9700
N2A—H2A	0.94 (4)	C18A—H18A	0.9700
N2A—C15A	1.493 (3)	C18A—H18B	0.9700
N2A—C16A	1.492 (3)	C18A—C19A	1.499 (4)
N2A—C18A	1.499 (3)	C19A—H19A	0.9300
C1A—H1A	0.9800	C19A—C20A	1.319 (4)
C1A—C2A	1.524 (3)	C20A—H20A	0.9300
C1A—C8A	1.525 (3)	C20A—C21A	1.472 (4)
C2A—C3A	1.392 (4)	C21A—C22A	1.401 (4)
C2A—C7A	1.398 (4)	C21A—C26A	1.393 (4)
C3A—H3A	0.9300	C22A—H22A	0.9300
C3A—C4A	1.395 (4)	C22A—C23A	1.379 (4)
C4A—H4A	0.9300	C23A—H23A	0.9300
C4A—C5A	1.366 (4)	C23A—C24A	1.395 (5)
C5A—C6A	1.381 (5)	C24A—H24A	0.9300
C6A—H6A	0.9300	C24A—C25A	1.383 (5)
C6A—C7A	1.386 (4)	C25A—H25A	0.9300
C7A—H7A	0.9300	C25A—C26A	1.393 (4)
C8A—C9A	1.387 (4)	C26A—H26A	0.9300
C8A—C13A	1.394 (4)	O1B—C1B	1.270 (4)
C9A—H9A	0.9300	O2B—C1B	1.223 (4)
C9A—C10A	1.395 (4)	N1B—C4B	1.329 (6)
C10A—H10A	0.9300	N1B—C5B	1.311 (5)
C10A—C11A	1.366 (5)	C1B—C2B	1.515 (4)
C11A—C12A	1.377 (5)	C2B—C3B	1.388 (4)
C12A—H12A	0.9300	C2B—C6B	1.392 (4)
C12A—C13A	1.385 (4)	C3B—H3B	0.9300
C13A—H13A	0.9300	C3B—C4B	1.389 (6)
C14A—H14A	0.9700	C4B—H4B	0.9300
C14A—H14B	0.9700	C5B—H5B	0.9300
C14A—C15A	1.512 (4)	C5B—C6B	1.379 (5)
C15A—H15A	0.9700	C6B—H6B	0.9300
C15A—H15B	0.9700		
			
C14A—N1A—C1A	113.4 (2)	H15A—C15A—H15B	108.0
C14A—N1A—C17A	107.66 (19)	N2A—C16A—H16A	109.6
C17A—N1A—C1A	109.43 (19)	N2A—C16A—H16B	109.6
C15A—N2A—H2A	104 (2)	N2A—C16A—C17A	110.1 (2)
C15A—N2A—C18A	110.1 (2)	H16A—C16A—H16B	108.2
C16A—N2A—H2A	113 (2)	C17A—C16A—H16A	109.6
C16A—N2A—C15A	109.35 (19)	C17A—C16A—H16B	109.6
C16A—N2A—C18A	112.1 (2)	N1A—C17A—C16A	111.3 (2)
C18A—N2A—H2A	108 (2)	N1A—C17A—H17A	109.4
N1A—C1A—H1A	108.0	N1A—C17A—H17B	109.4
N1A—C1A—C2A	110.3 (2)	C16A—C17A—H17A	109.4
N1A—C1A—C8A	112.5 (2)	C16A—C17A—H17B	109.4
C2A—C1A—H1A	108.0	H17A—C17A—H17B	108.0
C2A—C1A—C8A	110.0 (2)	N2A—C18A—H18A	109.1
C8A—C1A—H1A	108.0	N2A—C18A—H18B	109.1
C3A—C2A—C1A	121.8 (2)	H18A—C18A—H18B	107.8
C3A—C2A—C7A	118.5 (2)	C19A—C18A—N2A	112.7 (2)
C7A—C2A—C1A	119.6 (2)	C19A—C18A—H18A	109.1
C2A—C3A—H3A	119.6	C19A—C18A—H18B	109.1
C2A—C3A—C4A	120.7 (3)	C18A—C19A—H19A	118.8
C4A—C3A—H3A	119.6	C20A—C19A—C18A	122.3 (3)
C3A—C4A—H4A	120.6	C20A—C19A—H19A	118.8
C5A—C4A—C3A	118.7 (3)	C19A—C20A—H20A	115.9
C5A—C4A—H4A	120.6	C19A—C20A—C21A	128.3 (3)
F1A—C5A—C4A	119.2 (3)	C21A—C20A—H20A	115.9
F1A—C5A—C6A	118.0 (3)	C22A—C21A—C20A	122.7 (3)
C4A—C5A—C6A	122.8 (3)	C26A—C21A—C20A	119.1 (2)
C5A—C6A—H6A	121.0	C26A—C21A—C22A	118.1 (3)
C5A—C6A—C7A	117.9 (3)	C21A—C22A—H22A	119.6
C7A—C6A—H6A	121.0	C23A—C22A—C21A	120.7 (3)
C2A—C7A—H7A	119.3	C23A—C22A—H22A	119.6
C6A—C7A—C2A	121.4 (3)	C22A—C23A—H23A	119.8
C6A—C7A—H7A	119.3	C22A—C23A—C24A	120.4 (3)
C9A—C8A—C1A	119.3 (2)	C24A—C23A—H23A	119.8
C9A—C8A—C13A	119.4 (3)	C23A—C24A—H24A	120.1
C13A—C8A—C1A	121.3 (2)	C25A—C24A—C23A	119.7 (3)
C8A—C9A—H9A	119.7	C25A—C24A—H24A	120.1
C8A—C9A—C10A	120.6 (3)	C24A—C25A—H25A	120.2
C10A—C9A—H9A	119.7	C24A—C25A—C26A	119.6 (3)
C9A—C10A—H10A	120.9	C26A—C25A—H25A	120.2
C11A—C10A—C9A	118.3 (3)	C21A—C26A—C25A	121.4 (3)
C11A—C10A—H10A	120.9	C21A—C26A—H26A	119.3
F2A—C11A—C10A	119.1 (3)	C25A—C26A—H26A	119.3
F2A—C11A—C12A	118.1 (3)	C5B—N1B—C4B	116.4 (3)
C10A—C11A—C12A	122.8 (3)	O1B—C1B—C2B	113.8 (3)
C11A—C12A—H12A	120.7	O2B—C1B—O1B	126.3 (3)
C11A—C12A—C13A	118.6 (3)	O2B—C1B—C2B	119.9 (3)
C13A—C12A—H12A	120.7	C3B—C2B—C1B	122.3 (3)
C8A—C13A—H13A	119.9	C3B—C2B—C6B	116.9 (3)
C12A—C13A—C8A	120.3 (3)	C6B—C2B—C1B	120.8 (2)
C12A—C13A—H13A	119.9	C2B—C3B—H3B	120.6
N1A—C14A—H14A	109.7	C2B—C3B—C4B	118.9 (3)
N1A—C14A—H14B	109.7	C4B—C3B—H3B	120.6
N1A—C14A—C15A	110.0 (2)	N1B—C4B—C3B	124.0 (3)
H14A—C14A—H14B	108.2	N1B—C4B—H4B	118.0
C15A—C14A—H14A	109.7	C3B—C4B—H4B	118.0
C15A—C14A—H14B	109.7	N1B—C5B—H5B	117.6
N2A—C15A—C14A	111.0 (2)	N1B—C5B—C6B	124.8 (4)
N2A—C15A—H15A	109.4	C6B—C5B—H5B	117.6
N2A—C15A—H15B	109.4	C2B—C6B—H6B	120.5
C14A—C15A—H15A	109.4	C5B—C6B—C2B	119.0 (3)
C14A—C15A—H15B	109.4	C5B—C6B—H6B	120.5
			
F1A—C5A—C6A—C7A	−177.8 (2)	C14A—N1A—C1A—C8A	−44.2 (3)
F2A—C11A—C12A—C13A	179.6 (3)	C14A—N1A—C17A—C16A	61.8 (3)
N1A—C1A—C2A—C3A	30.7 (3)	C15A—N2A—C16A—C17A	54.7 (3)
N1A—C1A—C2A—C7A	−153.4 (2)	C15A—N2A—C18A—C19A	175.0 (2)
N1A—C1A—C8A—C9A	112.3 (3)	C16A—N2A—C15A—C14A	−55.8 (3)
N1A—C1A—C8A—C13A	−69.0 (3)	C16A—N2A—C18A—C19A	53.1 (3)
N1A—C14A—C15A—N2A	60.2 (3)	C17A—N1A—C1A—C2A	72.5 (3)
N2A—C16A—C17A—N1A	−59.2 (3)	C17A—N1A—C1A—C8A	−164.4 (2)
N2A—C18A—C19A—C20A	−116.8 (3)	C17A—N1A—C14A—C15A	−61.5 (3)
C1A—N1A—C14A—C15A	177.2 (2)	C18A—N2A—C15A—C14A	−179.3 (2)
C1A—N1A—C17A—C16A	−174.5 (2)	C18A—N2A—C16A—C17A	177.0 (2)
C1A—C2A—C3A—C4A	175.8 (2)	C18A—C19A—C20A—C21A	−179.0 (2)
C1A—C2A—C7A—C6A	−175.5 (2)	C19A—C20A—C21A—C22A	0.9 (4)
C1A—C8A—C9A—C10A	178.0 (3)	C19A—C20A—C21A—C26A	179.0 (3)
C1A—C8A—C13A—C12A	−178.2 (3)	C20A—C21A—C22A—C23A	177.5 (3)
C2A—C1A—C8A—C9A	−124.4 (3)	C20A—C21A—C26A—C25A	−177.2 (3)
C2A—C1A—C8A—C13A	54.3 (3)	C21A—C22A—C23A—C24A	−0.5 (5)
C2A—C3A—C4A—C5A	−0.1 (4)	C22A—C21A—C26A—C25A	1.0 (4)
C3A—C2A—C7A—C6A	0.5 (4)	C22A—C23A—C24A—C25A	1.3 (5)
C3A—C4A—C5A—F1A	178.1 (2)	C23A—C24A—C25A—C26A	−0.9 (5)
C3A—C4A—C5A—C6A	−0.1 (4)	C24A—C25A—C26A—C21A	−0.3 (5)
C4A—C5A—C6A—C7A	0.4 (4)	C26A—C21A—C22A—C23A	−0.6 (4)
C5A—C6A—C7A—C2A	−0.7 (4)	O1B—C1B—C2B—C3B	−171.0 (3)
C7A—C2A—C3A—C4A	−0.1 (4)	O1B—C1B—C2B—C6B	11.0 (4)
C8A—C1A—C2A—C3A	−93.9 (3)	O2B—C1B—C2B—C3B	9.0 (4)
C8A—C1A—C2A—C7A	82.0 (3)	O2B—C1B—C2B—C6B	−169.0 (3)
C8A—C9A—C10A—C11A	0.2 (4)	N1B—C5B—C6B—C2B	0.4 (6)
C9A—C8A—C13A—C12A	0.5 (4)	C1B—C2B—C3B—C4B	−178.9 (3)
C9A—C10A—C11A—F2A	−179.8 (3)	C1B—C2B—C6B—C5B	177.5 (3)
C9A—C10A—C11A—C12A	0.5 (5)	C2B—C3B—C4B—N1B	2.7 (6)
C10A—C11A—C12A—C13A	−0.7 (5)	C3B—C2B—C6B—C5B	−0.6 (5)
C11A—C12A—C13A—C8A	0.1 (5)	C4B—N1B—C5B—C6B	1.2 (6)
C13A—C8A—C9A—C10A	−0.7 (4)	C5B—N1B—C4B—C3B	−2.8 (6)
C14A—N1A—C1A—C2A	−167.3 (2)	C6B—C2B—C3B—C4B	−0.8 (4)

### Hydrogen-bond geometry (Å, º)

**Table:**

*D*—H···*A*	*D*—H	H···*A*	*D*···*A*	*D*—H···*A*
N2*A*—H2*A*···O1*B*^i^	0.94 (4)	1.62 (4)	2.557 (3)	176 (4)
C6*A*—H6*A*···O2*B*^ii^	0.93	2.60	3.439 (4)	151

Symmetry codes: (i) *x*, −*y*+1, *z*−1/2; (ii) *x*−1, *y*+1, *z*.
